# Stress Prevention@Work: a study protocol for the evaluation of a multifaceted integral stress prevention strategy to prevent employee stress in a healthcare organization: a cluster controlled trial

**DOI:** 10.1186/s12889-017-4585-0

**Published:** 2017-07-17

**Authors:** Rianne J. A. Hoek, Bo M. Havermans, Irene L. D. Houtman, Evelien P. M. Brouwers, Yvonne F. Heerkens, Moniek C. Zijlstra-Vlasveld, Johannes R. Anema, Allard J. van der Beek, Cécile R. L. Boot

**Affiliations:** 1 Department of Public and Occupational Health, Amsterdam Public Health research institute, VU University Medical Center, Van der Boechorststraat 7, 1081 BT Amsterdam, The Netherlands; 2 Body@Work TNO VUmc, Research Center on Physical Activity, Work and Health, VU University Medical Center, Van der Boechorststraat 7, 1081 BT Amsterdam, The Netherlands; 3 Department Occupation & Health, HAN University of Applied Sciences, Kapittelweg 33, 6525 EN Nijmegen, The Netherlands; 4 School of Social and Behavioural Sciences, Tranzo, Tilburg University, Warandelaan 2, 5037 AB Tilburg, The Netherlands; 5 Trimbos-institute, Netherlands Institute of Mental Health and Addiction, Da Costakade 45, 3521 VS Utrecht, The Netherlands; 6 Netherlands Organization for Applied Scientific Research TNO, Schipholweg 77-89, 2316 ZL Leiden, The Netherlands

**Keywords:** Implementation strategy, Work-related stress, Cluster controlled trial, E-health, Healthcare, Work-related stress management interventions

## Abstract

**Background:**

Adequate implementation of work-related stress management interventions can reduce or prevent work-related stress and sick leave in organizations. We developed a multifaceted integral stress-prevention strategy for organizations from several sectors that includes a digital platform and collaborative learning network. The digital platform contains a stepwise protocol to implement work-related stress-management interventions. It includes stress screeners, interventions and intervention providers to facilitate access to and the selection of matching work-related stress-management interventions. The collaborative learning network, including stakeholders from various organizations, plans meetings focussing on an exchange of experiences and good practices among organizations for the implementation of stress prevention measures. This paper describes the design of an integral stress-prevention strategy, Stress Prevention@Work, and the protocol for the evaluation of: 1) the effects of the strategy on perceived stress and work-related outcomes, and 2) the barriers and facilitators for implementation of the strategy.

**Methods:**

The effectiveness of Stress Prevention@Work will be evaluated in a cluster controlled trial, in a large healthcare organization in the Netherlands, at six and 12 months. An independent researcher will match teams on working conditions and size and allocate the teams to the intervention or control group. Teams in the intervention group will be offered Stress Prevention@Work. For each intervention team, one employee is responsible for applying the strategy within his/her team using the digital platform and visiting the collaborative learning network. Using a waiting list design, the control group will be given access to the strategy after 12 months. The primary outcome is the employees’ perceived stress measured by the stress subscale of the Depression, Anxiety, and Stress Scale (DASS-21). Secondary outcome measures are job demands, job resources and the number of preventive stress measures implemented at the team level. Alongside the trial, a process evaluation, including barriers and facilitators of the implementation of Stress Prevention@Work, will be conducted in one healthcare organisation.

**Discussion:**

If Stress Prevention@Work is found to be effective in one healthcare organisation, further implementation on a broader scale might lead to increased productivity and decreased stress and sick leave in other organizations. Results are expected in 2018.

**Trial registration:**

NTR5527. Registered 7 Dec 2015.

## Background

In Europe, one in four employees experience work-related stress [1]. Work-related stress can adversely influence health and cause a substantial amount of sick leave in organizations. Common mental disorders related to work-related stress, such as depression and anxiety, contribute the most to this sick leave [2, 3]. For example, the percentage of total number of sick leave days caused by psychological complaints, being stressed out, or being burned out was 22% in the Netherlands in 2015 [4].

Organizational work-related stress management interventions have shown positive effects on both sick leave and productivity [5, 6]. In addition, organizational work-related stress management interventions have shown positive effects on employee outcomes, such as confidence, coping, general health, and job satisfaction [7–10]. In addition to organizational interventions, stress management interventions on an individual level (e.g., a mindfulness program or assertiveness training) have shown positives effects on employees’ mental health, such as perceived stress, emotional exhaustion and anxiety state [10, 11]. Given the body of evidence, it is surprising that work-related stress management interventions are rarely used by organizations [12], in particular since the costs related to the consequences of work-related stress are high.

The key issue appears to be an implementation problem. Given the large number of available stress prevention strategies available on search engines such as google, information overload is likely. This results in difficulty selecting an intervention that fits well within an organization. Previous studies have shown that barriers associated with the implementation of work-related stress management interventions in organizations are present on three levels: sector, organization and employee [13]. To overcome implementation barriers at various levels across organisations, a combination of strategies has proven to be more effective than a single strategy [14, 15]. Therefore, we have developed a multifaceted, integral stress prevention strategy, Stress Prevention@Work (SP@W), to facilitate the selection and use of interventions in organizations. SP@W consists of two elements: 1) a digital platform (DP), which is an information technology platform containing a stepwise protocol to select and implement work-related stress management interventions, and 2) a collaborative learning network. The DP facilitates access to and the selection of matching work-related stress management interventions (evidence-based and/or practice-based). Interventions can be tailored to the specific needs of the team or organization either by someone within the organization or by an external coach or an implementation specialist. In previous research, a stepwise protocol has been shown to be effective by providing a framework for organizations that can be used to make suitable choices for work-related stress management interventions [16–18]. In addition to the DP, we initiated a collaborative learning network that provides contact between organizations to allow and facilitate the exchange of lessons learnt during the implementation of work-related stress management interventions. The network builds on earlier research showing that for the selection of suitable interventions, organizations often rely on a limited network of peer organizations for information about interventions [15, 19].

This paper describes SP@W and the protocol for the evaluation of SP@W by a cluster controlled trial, including an intervention and control group. We hypothesize that SP@W will decrease the employees’ perceived stress by reducing implementation barriers and increasing the implementation of work-related stress management interventions. A process evaluation will be guided by the framework of the Nielsen & Randall model for process evaluation of organizational interventions [20], while taking into account criteria described by Steckler and Linnan [21] that are relevant to the implementation process of SP@W, including barriers and facilitators.

## Methods

First, we describe SP@W, and next the protocol for the effect and process evaluation of SP@W.

### Integral stress prevention strategy Stress Prevention@Work (SP@W)

SP@W consists of two elements: 1) a digital platform (DP) with a stepwise protocol to select and implement work-related stress management interventions, and 2) a collaborative learning network. This two-element strategy was developed in close collaboration with several organizations from various sectors, including the healthcare sector. This participatory format enabled tailoring of the strategy to the needs of organizations. This is necessary because work-related stress has a wide range of determinants [8, 22]. Both elements of SP@W are described below.

### Digital platform (DP)

The DP consists of a stepwise protocol, multiple interventions, and screening instruments. The stepwise protocol consists of with five steps: 1) awareness, 2) assessment, 3) prioritizing and planning, 4) implementation, and 5) evaluation. The interventions are either at organizational or employee level with a focus on: online or offline groups or individuals; primary or secondary prevention, or specific sectors and organization types (e.g. small vs. medium-sized enterprises). An example of an offline organizational intervention is a guideline to start a dialogue between employees and their manager(s) about the presence of work-related stress within the organization or team [23]. An example of an individual online intervention is a self-help module to reduce work-related psychosocial risk factors [24]. Additionally, the DP provides screening instruments for (work-related) stress and psychosocial risk factors, and contains references to intervention providers who can assist in the implementation process if needed.

#### Development of the digital platform

We piloted the DP in five organizations in the educational, healthcare, transport and ICT sector aiming to achieve the optimization of the DP for use in multiple sectors. Supervisors, managers and Human Resource (HR) managers participated in these sessions. At the end of each session, a structured interview was performed about several aspects of the usability of the DP within their organisation, such as feasibility and accessibility, attitude towards the DP, motivation for use, financial feasibility, barriers, facilitators, and satisfaction. Based on these sessions, we optimized the DP to ensure that the steps of the DP’s stepwise protocol were easy to follow for the target group of HR managers in medium or large organizations.

#### The stepwise protocol

The first step focuses on evaluating the awareness and commitment the organisation. The second step focuses on finding or confirming the main work-related psychosocial risk factors of stress within a team and prioritizing the risks that will be tackled. The third step focuses on choosing the appropriate intervention(s). The fourth step focuses on implementing the chosen work-related stress management intervention(s), and the fifth step focuses on the evaluation of the process and effects, and evaluates if further interventions are necessary. The steps are described below.

##### Step 1 Awareness of stress in the workplace

Step 1 focuses on evaluating the awareness and level of stress present at the workplace, within a team, or in the organization using a multiple-choice question “*Is there stress at your workplace or in your organization?”* The answer categories are: “*Yes, there is stress at the workplace, we have an accurate idea of the problems”, “Yes, there is stress at the workplace, however we have no accurate idea of the problems”, “We do not know if there is stress at our workplace”,* and “*No, there is no stress at our workplace, but we want to focus on prevention of stress.”* The DP contains corresponding background information for this step focusing on indications of work-related stress and statistics of work-related stress across various sectors in the Netherlands.

##### Step 2 Problem assessment

Step 2 has a checklist with work-related psychosocial risk factors, presented in themes (Table 1). A priority level is chosen for each risk factor: “high priority”, “low priority”, “unknown”, or “no problem”*.* The checklist is based on literature about psychosocial risks and work-related stress. A preliminary list was discussed during a project meeting with all researchers involved in the development of the SP@W until consensus about the checklist was reached.

**Table 1:** Work-related psychosocial risk factors of stress, consequences and personal characteristics discussed on the digital platform

Themes	Examples
Psychosocial risk factors at work	
Job demands	Quantitative job demands, emotional requirements and cognitive load at work
Decision latitude and control	Task variety and autonomy
Social support	Supervisor and co-worker support
Violence and harassment	Violence, harassment and bullying—internal by colleagues or management or third party by clients, patients or the public
Job security	Precarious work, restructuring, temporary employment contracts
Consequences	
Work-life balance	Work-home interference and home-work interference
Health and resilience	Burnout or stress complaints and general health
Satisfaction	Job satisfaction
Performance and absence	Absenteeism, presenteeism, personnel turnover and productivity
Personal characteristics	
Personal resources	Coping style (active/passive)
Qualification	Education level, skills level

The DP provides background information on how to prioritize the work-related psychosocial risk factors and how to involve management or other team members in the identification and prioritization of psychosocial risk factors at work. It also provides suggestions on how to use information gathered earlier about work-related stress in the organization (e.g., an employee satisfaction survey). A questionnaire can be distributed within the team to measure work-related stress more accurately, if needed.

##### Step 3 Deciding on interventions

Based on the priorities identified in step 2, one or more work-related psychosocial risk factors are selected in step 3. The intervention teams then decide on the number and type of intervention(s) for implementation. A search engine connects the selected work-related psychosocial risk factors to matching stress management interventions. The search engine contains a short description of each intervention, including information about the type of intervention (e.g. individual or team programs) and the costs (“free of charge” or “costs involved”). In addition to the interventions provided by the search engine, interventions already available in the participating organization or a tailored intervention offered by a consultant are also available for selection. Apart from choosing an intervention or a consultant to provide a tailored-made intervention, good practice examples are available on how other organisations tackled psychosocial risks. Background information in this step focuses on how to make an action plan for the implementation of the stress management intervention and how to involve the employees.

##### Step 4 Implementation

In step 4, an action plan to implement the work-related stress management intervention(s) (selected in step 3) is made and the intervention is implemented. This plan includes who does what at which time in order to implement the intervention as planned. This step offers background information on how to foster good communication about the implementation of an intervention with the employees and/or management, and how to keep track of the implementation process, for example, by asking the employees how they experience the implementation of the intervention.

##### Step 5 Evaluation

Step 5 focuses on the evaluation of the process and effect of the implemented stress-management intervention using questionnaires. This information informs the organisation about what went well, and which parts should be adjusted in the future. The process evaluation questionnaire includes questions about three topics: 1) satisfaction with the results and process (e.g. “How satisfied are you [up to now] with the implementation process of the intervention?”), 2) factors for success and improvement, such as experienced support (e.g. “How satisfied are you with the support from the organisation during the implementation process?”), and 3) an open-ended question about changes during the implementation period (“Did the action plans (step 4) change during the execution of this project?”). The first two questions are answered using a 10-point Likert Scale. A report is automatically generated and includes an overview of the results. The effect evaluation is a checklist that is similar to the work-related psychosocial risk factors checklist in step 2 *‘Assessment of the problems’*. By completing this checklist, the stepwise protocol can be continued with a new round. Background information available in step 5 involves advice about evaluation methods.

### Collaborative learning network

The second element of SP@W is the collaborative learning network. The collaborative learning network aims to stimulate communication between organizations in enabling, sharing, and exchanging knowledge about implementation experiences and discussing good practices about implementing a stress-management intervention, and case histories for implementation between organizations. The aim of this network is that organization may learn from the other organizations, but may also be stimulated and energized by other organizations. Three learning networks were set up in three regions in the Netherland, including stakeholders from 20 distinct organizations from several sectors. We aim to organize three learning-network meetings over the trial one-year trial period, across all three regions, and one annual general meeting for all geographic regions together. Each meeting is approximately two hours, and addresses, e.g., one specific step from the stepwise protocol guided by the specific needs of the participating organizations. The meetings are chaired by an implementation expert.

### Effect and process evaluation of Stress Prevention@Work

#### Study design and measurements

The effectiveness of SP@W will be evaluated in a cluster controlled trial. The study population consists of employees of a Dutch healthcare organization. This organization has more than 4000 employees throughout the Netherlands, who are organized in self-directing teams. The organization facilitates care in nursing homes (including geriatric rehabilitation care), residential care homes, and home-based care. SP@W will be implemented in intervention teams during the first six months of the trial period. After the final follow-up measurement (12 months after baseline), SP@W will also be offered to the control teams. Digital questionnaires will be sent to employees from both the intervention and control teams at baseline and after six and 12 months to measure the short- and long-term effectiveness of SP@W. To maximize the response rate, two reminders will be sent to the employees for each questionnaire. The questionnaires will be in Dutch. The SP@W implementation process will be assessed by performing a process evaluation. The study procedures and design have been approved by the Medical Ethics Committee of the VU University Medical Center, Amsterdam, The Netherlands (2015.480).

### Recruitment, and inclusion and exclusion criteria

Recruitment of employees from a healthcare organization will involve a stepwise procedure. First, the management will decide which unit of the organisation will participate in the trial. Next, team coaches from the participating unit will be asked to select eligible teams to participate in the trial. Eligibility implies that the teams are willing to participate in the trial and able to provide a team member who will be responsible for the implementation of SP@W within the team during the trial period. A Human Resource (HR) manager or the team coach can also choose to take responsibility for implementing the SP@W within a team. Second, the employees within the eligible teams will receive an e-mail with the study information, inclusion and exclusion criteria, and the baseline questionnaire. The inclusion criteria are a minimum age of 18 years and an employment contract at the healthcare organization. Exclusion criteria are sick leave of more than one month at the time of inclusion or planned retirement within one year. Informed consent for participation in this study will be retrieved through an opt-in construction in accordance with the Dutch law on Medical Research in Humans. By completing the baseline questionnaire, eligible workers declare their agreement to participate in scientific research.

### Allocation and matching teams

After the baseline measurement, teams will be matched on their specific work setting (e.g. home-based care or nursing home care) and team size. The aim is to have both comparable teams and an equal number of participants in the intervention and control condition. This information will be obtained from the team coaches. Following the match, allocation to the two conditions will be performed by an independent researcher who does not have information about the perceived stress levels in the teams. After allocation, the teams are informed about the condition they are assigned to: intervention or control (waiting list) condition. A secure login code for the digital platform (DP) for all intervention teams will be sent to the employees responsible for applying SP@W within their team. The use of the DP will be explained during a training by an implementation expert. An invitation for this training will be sent to all responsible employees for the intervention teams. They will also receive an invitation for collaborative learning network meetings and the contact information of all employees responsible for the DP in the intervention teams.

### Effect evaluation

#### Primary outcome

The primary outcome stress will be measured by the stress subscale of the Depression, Anxiety and Stress Scale (DASS-21) at employee level [25]. The stress subscale consists of seven questions with a score range of 0–21. These questions relate to the experience of symptoms in the past week. The stress subscale measures the level of arousal. Examples of questions are “I found that I was very irritable”, “I found myself getting impatient when I was delayed in any way (e.g. lifts, traffic lights, being kept waiting)”, and “I found it difficult to relax”. The answering categories were “Did not apply to me at all”, “Applied to me to some degree, or some of the time”, “Applied to me to a considerable degree, or a good part of time”, and “Applied to me very much, or most of the time”. The internal consistency of the scale was good (Cronbach’s α 0.85). Construct validity and criterion validity of the DASS-21 have been measured before, and the criterion validity was good [25].

#### Secondary outcomes

##### Work-related psychosocial risk factors

Psychological job demands, supervisor support, co-worker support, and decision authority will be measured at employee level using four subscales from the validated Dutch version of the Job Content Questionnaire [26]. This questionnaire measures the physical and psychological characteristics of an imbalance between job demands and resources within an organization. The questions contain a four-point scale containing the options “Totally disagree”, “Disagree”, “Agree”, and “Totally agree”. The range is 5–20 for psychological job demands (five items), 4–16 for supervisor support and co-worker support (both 4 items), and 3–12 for decision authority (three questions).

##### Psychosocial Safety Climate

The Psychosocial Safety Climate (PSC) is measured at employee level, using the Dutch translation of the PSC-12, a 12-item questionnaire that includes four topics: 1) management support for employees’ psychological health, 2) management health and safety priorities, 3) organizational interest for employee participation in health and safety issues, and 4) communication of the organization to the employees about psychological health issues [27]. The questions are answered on a 5-point Likert scale that ranges from 1 (“Strongly disagree”) to 5 (“Strongly agree”). The scale score of the PSC-12 is the sum of all 12 questions ranging from 12 to 60. The PSC-12 will be adapted for this study to represent the organizational structure of the organization under study more accurately (e.g. we will change ‘supervisor’ to ‘team coach’, since this is the term used in the organization involved).

##### Sickness absenteeism and presenteeism

Sickness absenteeism (i.e., sick leave) and presenteeism will be measured at an employee level using the questionnaire on healthcare consumption and productivity loss in patients with a psychiatric disorder (TiC-P) [28]. Sickness absenteeism will be measured with one question: “Were you off work at any time in the past 4 weeks?” (“No”/“Yes, I missed … days of work” Presenteeism, will be measured using the question: “Was your job performance adversely affected by health problems during the past 4 weeks?” (“No”/“Yes, on how many days during the past four weeks did you perform paid work although you were bothered by health problems? …….. days”. If the answer is ‘yes’, the following two questions will be asked: 1) “Please rate how well you performed on the days you went to work even though you were bothered by health problems” This will be measured on a 10-point Likert Scale with 1 for “Much worse” to 10 for “performed as usual.” 2) “If you had to catch up on work you were not able to do due to health problems during the last 4 weeks, how many extra hours would you have to work?”

##### Implementation indicator

We aim to investigate implementation indicators to gain insight into the differences in the implementation of stress-management interventions between the intervention and control teams. First, we will collect information about the number of stress-management interventions implemented per team. Researchers will attend team meetings between the 6- and 12-month follow-up to ask the team about all interventions implemented since the start of SP@W. Second, we will include the following questions to the 12-month follow-up questionnaire: “*How often did you discuss work-related stress in your team (for example during a team meeting of your team)?”* and “*To what extent have work-related stress management interventions been implemented in your team since (month) (year)?”* The answers will be measured on a 10-point Likert Scale with 1 for “Not at all/barely” to 10 for “Very often/very much”. Both questions will be analysed at a team level to estimate an implementation score for each team.

#### Prognostic factors

Several prognostic factors will be investigated at baseline to gain insight into the differences between the intervention and control teams.

##### Sociodemographic measures

Gender (male/female), age (years), Dutch nationality (yes/no), marital status (married/registered partnership/living together; in a relationship/not living together; single/divorced/widow/widower), and the highest educational degree (no schooling completed/primary school; lower vocational education/ secondary vocational education; higher professional education/university) will all be measured at the employee level.

##### Work characteristics

The number of years working for the organization, working days and hours per week, and working at night (yes/no), during the weekend (yes/no) or evenings (yes/no) will all be measured at the employee level.

##### Healthcare consumption

Healthcare consumption is measured at the employee level using the questionnaire on healthcare consumption and productivity loss in patients with a psychiatric disorder (TiC-P) [28]. The number of times the participant consulted a healthcare provider (by phone or visiting during office hours) will be asked using the following questions: “Did you consult (*name healthcare provider*) at any time during the past four weeks?” (“No”/“Yes, … times”), and, if the answer is ‘yes’: “How many times were you in contact with the *healthcare provider* due to depressive complaints?” Healthcare consumption will be measured for the following healthcare providers: general practitioner, physiotherapist, practice nurse mental health, lifestyle counsellor, chiropractor, occupational therapist, exercise therapist, psychiatrist, psychologist, psychotherapist, social worker, and health and safety officer.

##### Co-interventions

We will collect information about all initiatives possibly related to stress prevention@work during the trial period through interviews with stakeholders within the organization.

### Process evaluation

Alongside the effectiveness evaluation, a process evaluation will be conducted. The Nielsen & Randall model for organizational interventions and the Steckler and Linnan framework for process evaluations will be used to gain insight into the implementation process of the SP@W strategy [20, 21]. The criteria described by Steckler and Linnan that are most relevant to the implementation goal are: ‘reach’ and ‘dose received’. Reach is the percentage of intervention teams that participated in SP@W. Dose received will be operationalized as the number of steps of the stepwise DP protocol that are completed. Both reach and dose received will be assessed at the team level. Data for the process evaluation will be collected using questionnaires completed by participants of the intervention teams, interviews with stakeholders, team and stakeholder meeting notes, and DP data logs.

### Sample size

A power analysis has been performed for the main outcome measure: the stress subscale of the DASS-21. This scale has a mean of 4.06 and a standard deviation of 3.81 in a non-clinical sample of North American adults [29]. With a power of 0.80 (1-beta) and an alpha of 0.05, we need 208 participants (104 per group) to demonstrate a relevant effect in the stress scale of 1.5 points. Taking into account a loss to follow-up of 25% after 12 months, a total of 278 employees need to be included.

### Statistical analyses

To investigate the effectiveness of SP@W, all analyses will be performed according to the intention-to-treat principle. Descriptive statistics (means, standard deviations, or frequencies) will be calculated for all measured variables, and will be compared between the intervention and control groups. The effects of SP@W will be analysed by performing a multilevel analysis, while taking into account clustering within teams. A two-tailed significance level of *p* < 0.05 will be considered statistically significant.

## Discussion

This paper described the study protocol of the multifaceted and integral stress prevention strategy, SP@W. It is hypothesized that after a successful implementation of SP@W, the strategy will increase the mean number of work-related stress management interventions implemented within the intervention teams compared to the control teams and lead to a decrease in employee stress over 12 months.

### Strengths and limitations

The first strength of this study is our controlled design. This allows for a comparison between teams within the participating healthcare organization who are exposed to similar environmental conditions that may lead to stress (e.g. organizational changes), if not for access to SP@W. The second strength of this study design is the follow-up period of one year, which allows insight into short-term effects of the intervention after six months and in the longer term after 12 months. Third, by incorporating a process evaluation, we will gain insight into the strategy’s implementation process within the intervention teams. Last, this study will be executed in a real-life setting, which will make it easier to generalize the effects to similar healthcare organizations.

Since SP@W focuses on the organization and not on individual workers, individual randomisation is not feasible. Matching controls may induce bias since matching a limited number of known parameters may lead to differences between teams in the intervention and control group that we are unaware of. Furthermore, we cannot completely rule out contamination since all teams belong to one healthcare organization. A cross-over of information can reduce the contrast between the groups and consequently, the impact of the effects.

### Impact of the results

A combination of several implementation strategies into one integral stress prevention strategy is important to overcome barriers at multiple levels within organizations. SP@W may lead to a reduction of stress and sick leave and an increase in productivity, which is beneficial for employees as well as for the organization.

## Abbreviations

DASS-21: Depression, anxiety and stress scale
DP: Digital platform
HR: Human resource
PSC: Psychosocial safety climate
SP@W: Stress prevention@work
